# Assembly of a phased diploid *Candida albicans* genome facilitates allele-specific measurements and provides a simple model for repeat and indel structure

**DOI:** 10.1186/gb-2013-14-9-r97

**Published:** 2013-09-11

**Authors:** Dale Muzzey, Katja Schwartz, Jonathan S Weissman, Gavin Sherlock

**Affiliations:** 1 Department of Cellular and Molecular Pharmacology, California Institute for Quantitative Biomedical Research, and Howard Hughes Medical Institute, University of California, San Francisco, San Francisco, CA 94158, USA; 2 Department of Genetics, Stanford University, Stanford, CA 94305, USA

**Keywords:** Haplotype, Phasing, Indel, Microsatellite, Homopolymer, Repeat

## Abstract

**Background:**

*Candida albicans* is a ubiquitous opportunistic fungal pathogen that afflicts immunocompromised human hosts. With rare and transient exceptions the yeast is diploid, yet despite its clinical relevance the respective sequences of its two homologous chromosomes have not been completely resolved.

**Results:**

We construct a phased diploid genome assembly by deep sequencing a standard laboratory wild-type strain and a panel of strains homozygous for particular chromosomes. The assembly has 700-fold coverage on average, allowing extensive revision and expansion of the number of known SNPs and indels. This phased genome significantly enhances the sensitivity and specificity of allele-specific expression measurements by enabling pooling and cross-validation of signal across multiple polymorphic sites. Additionally, the diploid assembly reveals pervasive and unexpected patterns in allelic differences between homologous chromosomes. Firstly, we see striking clustering of indels, concentrated primarily in the repeat sequences in promoters. Secondly, both indels and their repeat-sequence substrate are enriched near replication origins. Finally, we reveal an intimate link between repeat sequences and indels, which argues that repeat length is under selective pressure for most eukaryotes. This connection is described by a concise one-parameter model that explains repeat-sequence abundance in *C. albicans* as a function of the indel rate, and provides a general framework to interpret repeat abundance in species ranging from bacteria to humans.

**Conclusions:**

The phased genome assembly and insights into repeat plasticity will be valuable for better understanding allele-specific phenomena and genome evolution.

## Background

The advent of short-read DNA sequencing has resulted in super-exponential growth in the quantity of available sequencing data. Along with a dramatic increase in the number of assembled reference genomes for different species, much recent effort has been focused on defining the sequence variants - such as SNPs and insertions/deletions (‘indels’) - between individuals of the same species. The focus of many such studies is the resolution of haplotypes [1], which specify which variant bases are inherited together on contiguous DNA. Despite this interest in determining the phasing of polymorphisms, short read lengths complicate the resolution of haplotypes: adjacent polymorphisms must be sequenced in the same molecule to be included in the same haplotype. Several elegant approaches can overcome this difficulty, including the coupling of pedigree analysis with sequencing data [2,3] and, more recently, various methods of spatially partitioning whole homologous chromosomes - via microdissection [4], microfluidic device [5], or dilution [6-10] - such that they can be separately barcoded, amplified, and sequenced before assembly into barcode-defined haplotypes.

Most of the effort in haplotype discovery has focused on humans; thus, there are few phased genomes available in other multiploid model organisms. Knowing the phasing information in model organisms, however - for example, those that are single-celled, have compact genomes, double rapidly, and are easily manipulated genetically - is useful for a variety of reasons, including ease of measurement of allele-specific phenomena in different genetic backgrounds and observation of homolog-specific evolution on laboratory timescales. *Candida albicans* is a model fungal pathogen that almost exclusively exists in a diploid state and does not achieve genome diversity via a typical meiotic cycle with frequent recombination. Instead, it employs one of two strategies, both involving mating and whole chromosome loss, where the order of these events is inverted. First, in the so-called ‘parasexual cycle’ [11], two diploids of opposite mating type can mate to yield a tetraploid, and then return to the diploid state via chromosome loss, a process that can occasionally result in homozygosis of single chromosomes [12,13]. Alternatively, a recent report revealed that chromosome loss can occur first to generate a mating-competent haploid, which can subsequently mate to restore the diploid state [14]. Importantly, both mating options occur rarely in *C. albicans*, and both leave the homologs largely intact. Thus, the phasing of polymorphisms in *C. albicans* has fewer entropic, degenerating forces than in most other organisms, making the assembly of its phased genome particularly desirable.

**Figure 1:**
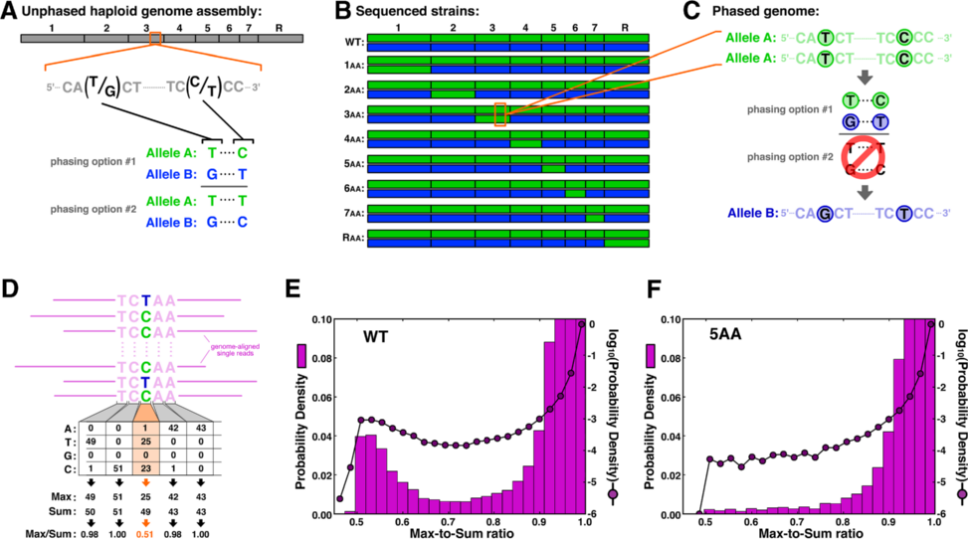
**Sequencing of strains that contain homozygous regions can resolve genome phasing. (A)** Schematic illustrating the ambiguous phasing of two adjacent SNPs from chromosome 3 of *C. albicans* genome Assembly 21. **(B)** Idealized panel of strains to resolve phasing. The wild-type (WT) strain is heterozygous for all eight chromosomes, having both the A homolog in green, and the B homolog in blue. Additional strains to sequence were selected to be homozygous for the indicated chromosomes. **(C)** One phasing option from **(A)** can be excluded by sequencing the ‘3AA’ strain, since all reads are effectively from the A allele, pairing the T and C; SNPs on the B allele are inferred. **(D)** Illustration of how to calculate the max-to-sum ratio, with a SNP position highlighted in orange. **(E**, **F)** Histograms of max-to-sum ratios for all positions across chromosome 5 in wild type **(E)** and the ‘5AA’ strain **(F)**; bars are in linear space, and the line plot is in log space.

Extensive sequencing of *C. albicans* and many closely related *Candida* species has yielded important insight into the pathogenicity of *C. albicans*[15], as well as a host of valuable whole-genome assemblies. The first official release of the *C. albicans* genome, Assembly 19, was partially diploid and identified thousands of polymorphisms using low-coverage Sanger sequencing, but had long haploid spans and did not assemble the genome into full chromosomes [16]. The next major release, Assembly 21 [17], assembled contigs into whole chromosomes but was a reftig-based assembly, that is, the alleles present within a given chromosome were equally likely to have originated from one of the two haplotypes (Figure 1A). The first attempt at phasing the genome involved using microarrays to probe 38,000 SNPs identified in the low-coverage assemblies [18]. Here we advance the genome-phasing effort by using next-generation sequencing. Our nearly 100-fold improvement in coverage relative to prior assemblies nearly doubles the number of high-confidence SNPs and indels that could be assigned to their respective homologs. This increase in phasing resolution within our diploid genome assembly permits more sensitive analysis of allele-specific phenomena and provides insight into genome architecture and evolution.

## Results

### SNP identification from deep sequencing of wild-type and homozygous strains

To resolve polymorphism phasing in *C. albicans*, we performed deep sequencing on genomic DNA prepared from a panel of nine strains, including wild-type SC5314 and eight related strains, each known to be homozygous for one of the eight *C. albicans* chromosomes (Figure 1B). Our approach involved three steps: identification of polymorphisms in the strains that are heterozygous for a chromosome, resolution of one of the haplotypes (that is, either the A or B homolog) via direct sequencing of the corresponding homozygous strain (Figure 1C, top), and finally inference of the sequence of the opposite haplotype (Figure 1C, bottom). The inference step for the opposite homolog was likely unavoidable, since certain chromosomes are thought to contain recessive lethal alleles and have not been observed in a homozygous state [14]. For each strain, we generated approximately 40 million reads (that is, paired-end reads of 20 million DNA fragments), with 36 nucleotides sequenced per read, giving approximately 100-fold coverage per base ((40e6 reads) × (36 nucleotides/read)/(14,324,316 nucleotides/genome) ~ 100). Since multiple strains are heterozygous for each chromosome, on average we had 700-fold coverage of heterozygous data for each base.

Since we had such high coverage at each position, we identified SNPs *de novo* from our sequencing data, without consideration of SNPs previously reported. We first aligned all reads - irrespective of their paired-end counterpart - to the Assembly 21 genome, using the three-mismatch maximum allowed by BowtieV1.0 [19]. We were concerned that a densely polymorphic region (that is, more than three SNPs in a 36 nucleotide window) could be spuriously reported as being devoid of SNPs since any read reporting >3 SNPs would fail to align to the haploid reference. To address this issue, for any read that initially failed to align, we checked to see if its paired end successfully aligned and, if so, searched within the empirical fragment size (200 to 800 nucleotides; Additional file 1: Figure S1) of the aligned position for a best match (Additional file 1: Figure S2A). For this best-match search, we allowed up to 6 mismatches (that is, we required ≥30 nucleotide perfect match) and found that approximately 50% of initially unaligned reads could now be mapped to the genome (Additional file 1: Figure S2B). This strategy dramatically increased the number of densely polymorphic regions we identified (Additional file 1: Figure S2C).

After aligning reads to the reference, we tabulated the number of counts for each base (that is, A,T,G,C) at each position across the genome (Figure 1D, top). Next, at each position we calculated the max-to-sum ratio, which is the maximum number of counts among the four bases divided by the sum of all counts (Figure 1D, bottom). Non-polymorphic positions are characterized by max-to-sum ratios near 1.0, whereas a typical SNP should have a max-to-sum ratio of approximately 0.5, assuming that the two homologs are sequenced comparably. Empirical data generated from the wild-type strain supported the use of the max-to-sum ratio in SNP identification: in a histogram of max-to-sum ratios for each position across chromosome 5, there was clear separation between the approximately 99.5% of positions that were non-polymorphic with max-to-sum ratios in excess of 0.9 (Figure 1E) and the approximately 0.5% with max-to-sum ratios near 0.5 (Figure 1E). To confirm that the peak near 0.5 was composed of heterozygous SNPs, we compiled a similar histogram for data from the ‘5AA’ strain, which is homozygous for chromosome 5 and found that the peak near 0.5 disappeared (Figure 1F).

**Figure 2:**
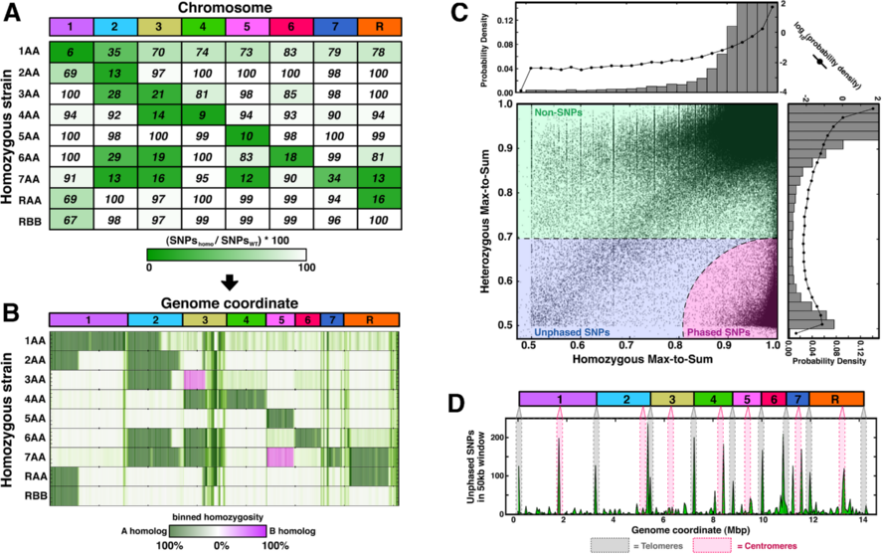
**Pooling reads across heterozygous and homozygous regions clearly identified SNPs. (A)** For each homozygous strain independently, the number of positions with max-to-sum ratio <0.7 were considered ‘putative SNPs’; the total number of putative SNPs on each chromosome was called SNPs_*homo*_, and this number was divided by the corresponding value for wild type; to avoid confusion, the plotted number is the minimum of this quotient and 100%. **(B)** Putative SNP locations were identified in the wild-type strain, and the corresponding positions in homozygous strains were investigated for SNP status: if a putative SNP position from wild type was not a SNP in the indicated strain, it was shaded green (or pink, depending on the allele), whereas if both were SNPs, the latter was shaded white. **(C)** Scatterplot of max-to-sum ratios in heterozygous and homozygous regions for every position in the genome. Histograms at top and right show the distribution of data on each perpendicular axis as indicated, with bars in linear space and lines in log space. **(D)** The number of unphased SNPs in non-overlapping 50 kb windows tiled across the genome, with telomere and centromere locations as indicated.

Regions of unexpected homozygosity enhanced our SNP-identification procedure. Our initial strategy (Figure 1B) assumed that the only homozygous regions in our panel of strains were the chromosomes selected to be homozygous. Indeed, when we compared the number of SNPs per chromosome identified in wild type versus each of the selected strains, we found that the chromosomes selected to be homozygous had very low SNP numbers (Figure 2A, dark-green shading on diagonal). However, we also observed considerably lower SNP density on chromosomes in many other strains (Figure 2A, off-diagonal green shading). When we looked further at the density of homozygosity as a function of position along the chromosome (Figure 2B), it became clear that many strains were homozygous not only for the selected chromosome, but also for other entire chromosomes, or megabase-scale segments of chromosomes. Appropriately specifying these unexpected regions of homozygosity - rather than simply implementing our strategy from Figure 1B - both avoided corruption of our SNP-finding signal in ostensibly heterozygous regions and enhanced our ability to resolve SNP phasing in homozygous regions.

The length-scale of our phasing results is on the order of whole chromosomes. Interestingly, for the entirety of chromosome 5 and nearly a megabase of chromosome 3, at least one strain was homozygous for the opposite homolog as the other(s) (Figure 2B, pink shading). In fact, for the entire length of chromosome 5, the ‘5AA’ and ‘7AA’ strains report perfectly opposed SNP phasing - for example, where the heterozygous data indicate an A/C SNP, the ‘5AA’ and ‘7AA’ strains report exclusively A and C, respectively. The absence of observing the opposite homolog for other chromosomes (for example, 2, 6, 7, R, and so on) is consistent with other reports suggesting that the opposite homologs contain recessive lethal mutations [14]. The fact that we did not observe frequent phase switching (that is, short spans of adjacent pink and green stretches) across chromosomes homozygous in multiple strains (for example, first half of chromosome 2) also suggests that the phasing persists for whole chromosomes and is not interrupted by random intermixing between homologs.

Across the whole genome, we identified a total of 69,688 SNPs and were able to phase 94.4% of them. After separately pooling homozygous and heterozygous counts at each position based on Figure 2B, we made a scatterplot of max-to-sum ratios (Figure 2C). For each base, we measured the Euclidean distance in max-to-sum ratio units from the lower-right corner (that is, *[1.0,0.5]*). Positions with distance <0.195 - where the marginal true- and false-positive rates are equal (Additional file 1: Figure S3) - were called phased SNPs. Positions outside of this boundary but with heterozygous max-to-sum ratio <0.695 were called unphased SNPs. We found that unphased SNPs were primarily confined to telomeric and centromeric regions (Figure 2D), consistent with high repeat density in these locations, which would compromise read alignment.

Our study appreciably revises and expands the number of SNPs in the laboratory standard *C. albicans* SC5314 strain. We succeeded in mapping 98.25% (54,858) of the previously identified SNP positions from the contig-based coordinates of Assembly 19 into the chromosomes of Assembly 21. Of these, 75% (41,298) were corroborated and phased in this study. The average heterozygous max-to-sum ratio of the remaining 25% that were not confirmed as SNPs was 0.92, strongly suggesting that these positions are not polymorphic but were perhaps misidentified as such due to the lower coverage of previous assemblies. The 69,688 total SNPs we identified here thus represent an increase of nearly 69% (69,688/41,298 ~ 1.69) in the number of known SNPs.

An independent test of phasing fidelity confirms our results. Since paired-end fragments necessarily originate from a contiguous DNA molecule, they are properly phased by definition. Thus, we assessed the validity of our phasing method - which treats each nucleotide position independently of all others - by determining the consistency in phasing across all SNP positions included in the 72 nucleotides (2 ends × 36 nucleotides/end = 72 nucleotides) sequenced in each set of paired-end reads. We found that 94% of SNPs were part of a paired-end molecule in which both ends had SNPs, suggesting that this assay to compare phasing at opposing paired ends was nearly exhaustive. Of these, 99.8% of SNPs were consistently phased between the two paired ends. Further, the molecules with phasing disparity between the two ends were highly localized in a few positions in the genome, nearly all corresponding to adhesion genes of the ALS family (for example, *ALS2*, *ALS4*, and *ALS9*), which are largely identical but divergent enough to complicate read-mapping and SNP resolution [20].

### SNP phasing facilitates detection of allele-specific effects

**Figure 3:**
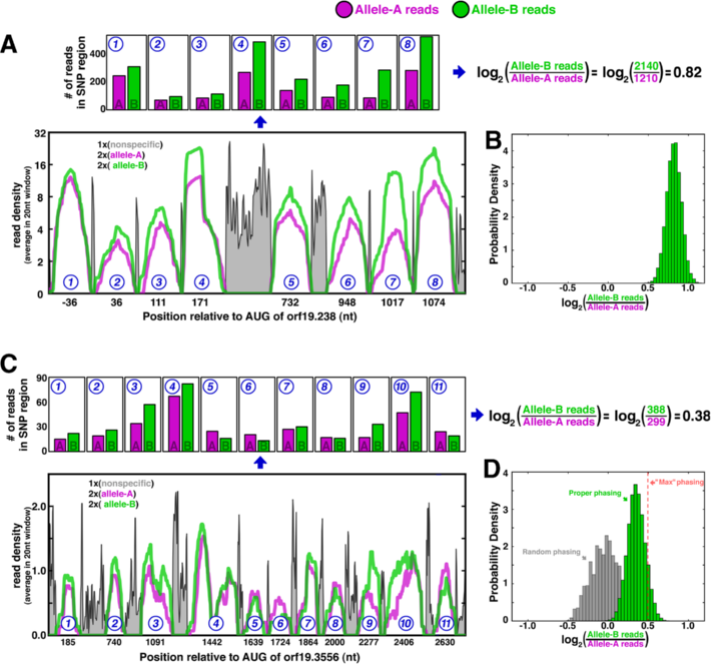
**Allele-specific bias in transcription is evident from pooling reads across phased SNPs. (A**, **C)** orf19.238 **(A)** and orf19.3556 **(C)** have 8 and 11 non-overlapping regions, respectively, where RNA-seq reads include SNPs and can be attributed to either allele A in purple, or allele B in green. The bar graphs at top quantify the number of reads per SNP region, with the line graph at bottom indicating read density in a 20 nucleotide sliding window across each region. The density of reads lacking SNP information is indicated in gray. For visual clarity, the x-axis is nonlinear, such that SNP regions show data at every nucleotide, and non-SNP regions show data every 10 nucleotides. **(B**, **D)** Allele-specific biases for orf19.238 **(B)** and orf19.3556 **(D)**, where histograms reflect the results from 10,000 bootstrap iterations. **(D)** The gray histogram shows how randomly permuting the phasing masks allele-specific bias, and the ‘max phasing’ line indicates the bias calculated if the maximum and minimum values for each bar in the top of **(C)** were attributed to allele B and allele A, respectively.

SNP phasing increases the precision with which allele-specific phenomena, such as allele-specific messenger RNA expression, can be measured. Using an RNA-seq dataset generated from the wild-type SC5314 strain grown in rich media [21], we mapped reads to our phased genome assembly. Reads that overlapped SNP positions were further interrogated to determine whether the SNP base corresponded to the A or B allele. The number of SNP-containing reads across the entire gene was summed based on their allelic origin, and the two allele-specific sums were compared. A representative gene, orf19.238, displaying a nearly two-fold allele-specific bias is shown in Figure 3A. There are eight distinct SNP windows across orf19.238, and the number of B-specific reads exceeds the A-specific reads across every SNP window (Figure 3A, top). This corroboration of bias across multiple SNPs in the same gene is a critical tool in assessing allele-specific effects [22-24] and was one of our primary motivations for increasing the phasing resolution over previous efforts [18]. In total, SNP-containing reads mapped to 427 different nucleotide positions across the gene, and since extreme count values at single positions could dominate the allele-specific signal, we used bootstrapping to determine a confidence interval in the fold-change measurement. In each of 10,000 simulations, we calculated the fold change of allele bias using counts from 427 positions selected randomly and with replacement from the empirical set of SNP-containing positions. This bootstrap analysis suggests that the fold-change difference is almost certainly in excess of 40% (Figure 3B) and is most likely 77% (2^0.82^ = 1.77).

By pooling reads across many SNPs, small but significant allele-specific biases are detectable. There are 11 SNP windows across orf19.3556, and summing reads across SNP windows indicates a 30% bias in expression of the B allele over the A allele (Figure 3C), with the entirety of the bootstrap distribution above zero fold change (Figure 3D). Bias toward the B allele is evident across seven of the SNP windows but not all: if allele-specific expression were calculated by pooling the maximum and minimum read counts, respectively, across all SNP windows, the result would overestimate the true allelic bias by almost 50% (Figure 3D, red line). As expected, in the absence of phasing information (Figure 3D, gray histogram) there is no allelic bias. Thus, the phased genome enables highly sensitive and accurate determination of allele-specific effects.

In addition to their utility in detecting allele-specific expression, certain SNPs can also cause allele-specific effects themselves. For instance, 198 alleles have premature termination codons (PTCs) relative to their partner alleles (see Additional file 2 for gene-by-gene characteristics in the phased assembly). PTCs are concentrated near the 5′ and 3′ ends of the coding sequence (Additional file 1: Figure S4), perhaps since alleles of intermediate length could yield dominant-negative proteins that confer a selective disadvantage. Since PTCs can elicit nonsense-mediated decay (NMD) [25], we investigated whether alleles with PTCs were less abundant in the RNA-seq dataset [21] than their counterpart alleles. Based on strict criteria (see Methods), we compiled a list of NMD-candidate genes and found that 73% (16/22) had an allelic expression bias of 20% or greater, and 75% (12/16) of those with a bias had fewer reads from the PTC-containing allele than from the allele without a PTC (Additional file 1: Table S1). These data are consistent with NMD, though further experiments would be required to establish this link conclusively.

### Indels accumulate in the repeat sequences of promoters

**Figure 4:**
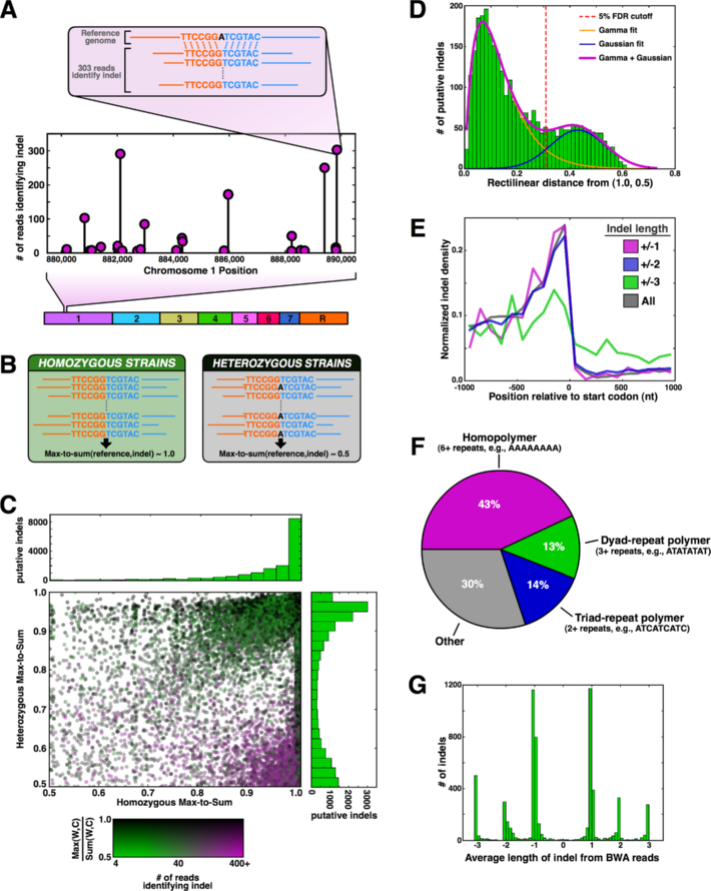
**Indels are enriched in repeat sequences upstream of genes. (A)** Close-up of 10 kb region of chromosome 1 containing several positions where hundreds of reads deviate from the reference in support of an indel. **(B)** Expected values for max-to-sum ratios of ‘reference’ and ‘indel’ reads in heterozygous and homozygous regions. **(C)** Scatterplot of max-to-sum ratios in heterozygous and homozygous regions for every putative indel in the genome. Histograms at top and right show the distribution of data on each perpendicular axis as indicated. The color of each point is based on the legend, where W and C indicate reads from the Watson and Crick strands, respectively. **(D)** The cutoff for indel designation, indicated in red, has a 5% false discovery rate (FDR), based on fitting the sum of gamma and Gaussian distributions, which reflect the true and false indels, respectively. The histogram in green considered only points with homozygous max-to-sum ratios <1.0 and rectilinear distances of 0.6 or less from the point *[1.0,0.5]*. **(E)** Indel density as a function of indel size and distance from the start codon. Density values were normalized to account for the fact that not all coding or intergenic regions span 1,000 nucleotides. **(F)** Indels are strongly enriched in repeat sequences. **(G)** Indels are not a sequencing artifact. The average size reported by all reads supporting an indel was calculated and then compiled into a histogram representing all indels. Random sequencing errors would have yielded density at non-integer values and, more importantly, around zero.

We identified and phased 6,103 short indels in the *C. albicans* genome. All reads that failed to align to the reference using software that disallowed gaps were later re-aligned using gap-permitting software (see Methods). We tabulated the gap positions into a genome-wide histogram, identifying thousands of putative indel positions in the genome, many with hundreds of reads supporting an indel (Figure 4A). As with SNPs, we separately considered reads from homozygous and heterozygous regions, designating reads as ‘reference’ if they matched the reference allele in Assembly 21 and ‘indel’ if they contained a gap relative to the reference. Our expectation was that a true indel should satisfy the following criteria: (1) have a max-to-sum ratio for reference versus indel counts near 1.0 in homozygous regions and 0.5 in heterozygous regions (Figure 4B), and (2) have a high number of reads comparably distributed between the Watson and Crick strands in support of the indel (Figure 4C). Since the rectilinear distance from *[1.0,0.5]* to each indel’s position in the scatter is effectively the sum of two exponentials - one each from the homozygous and heterozygous distributions (Figure 4C, top and right histograms) - the histogram of all such distances is well fit by a gamma distribution, with spurious background captured by addition of a Gaussian (Figure 4D). The cutoff distance for valid indels was chosen to yield a 5% false discovery rate, giving 6,103 indels in total. As with the phasing of SNPs, we independently validated the phasing of indels by ensuring that there was phasing coherence between paired-end reads where one end had an indel and the other had at least one indel or SNP.

Indels are not uniformly distributed across the genome and have a strong bias for repeat sequences. As expected, indels of size ±1 and ±2 are largely excluded from coding regions (Figure 4E), since these would disrupt the polypeptide reading frame. The same is not true of ±3 indels, which are only slightly depleted in coding versus noncoding regions. Strikingly, however, we observed strong enrichment for ±1 and ±2 indels in the first several hundred bases immediately upstream of coding regions, consistent with a higher rate of indels occurring in the regulatory regions of genes. In eukaryotes, these regulatory regions are populated with repeat sequences that help to exclude nucleosomes [26,27]. Consistent with other reports [28,29], we found that indels are highly enriched in repeat sequences (Figure 4F), which act as the substrate for nearly 70% of all indels identified.

Since indels in the repeats of promoters could affect gene expression via their role in nucleosome positioning, we tested whether indel density in the promoter correlated with allelic bias from the RNA-seq data [21], but we found no relationship. Two related factors could explain this lack of correlation: (1) short changes in repeat length (for example, the one-nucleotide and two-nucleotide indels that predominate in the *C. albicans* genome) are expected to yield minor expression effects (<20%) based on fluorescence reporter systems that isolated their relative contribution [27], and (2) rather than being isolated, the effect of indels in our assay for allelic bias is instead convolved with the allelic bias imparted by SNPs, which are required to detect allele-specific expression from RNA-seq data in the first place.

The indels identified were not artifacts of systematic errors in the sequencing of repeats. We plotted the average indel length reported by all reads at each validated indel position and summarized the results in a histogram (Figure 4G). If random errors in repeat-sequence length accounted for the observed indels, then we would expect a broad normal distribution centered at each integer value (with a peak also at length zero). However, we observed sharp peaks like delta functions at each integer, indicating that the hundreds of individual reads typically revealing each indel frequently all report the same indel length, supporting the validity of our indel identification.

### Indels cluster along the genome, especially near replication origins

**Figure 5:**
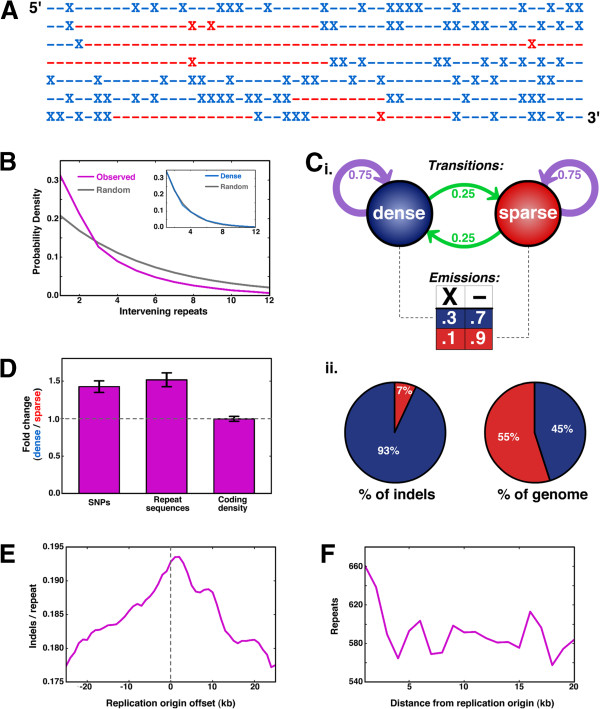
**Indels are clustered throughout the genome. (A)** A representative multikilobase span, where ‘X’ indicates an indel and dashes signify non-polymorphic repeat sequences. **(B)** The number of ‘–‘ characters between each indel (‘X’) was counted across the genome and compiled into a histogram in purple. In gray, the exponential distribution expected based on the observed indel probability and assuming random dispersion of indels. Inset: the analogous plot for ‘dense’ regions identified by the hidden Markov model (HMM). **(C)** (i) Schematic of the HMM used to distinguish indel-dense from indel-sparse regions. (ii) Fractional share of total indels (left) and number of bases in the genome (right) present in ‘dense’ (blue) and ‘sparse’ (red) regions. **(D)** Relative enrichment of three different sequence features between ‘dense’ and ‘sparse’ regions. Error bars indicate ±S.E.M. across regions, propagated through division. **(E)** The indel concentration, measured as indels-per-repeat sequence, in 7.5 kb windows centered at replication origins was calculated as a function of replication-origin offset (that is, 0 kb is the native origin location). Step size is 1 kb, and the average value across three adjacent windows is plotted. **(F)** The total number of repeat sequences present in non-overlapping 1 kb windows centered at replication origins.

Indels are not uniformly distributed throughout the genome. This clustering of indels is conspicuous in a genome-wide string indicating indel positions with an ‘X’ and each likely indel substrate (that is, mono-, di-, and tri-nucleotide repeats of length 8+) with a dash (‘–’ in Figure 5A). If ‘X’ positions were randomly scattered throughout this binary string, the separation of indels - measured in ‘–’ units - would be exponential, but we instead observed a kinked exponential curve (Figure 5B). To identify indel-dense regions systematically, we implemented a simple two-state hidden Markov model (‘HMM’; Figure 5C(i)), where the probability of indels in ‘dense’ regions is three times that in ‘sparse’ spans. We selected the HMM parameters such that indel spacing within dense regions was exponential (Figure 5B inset), suggesting that there are not additional levels of clustering in ‘dense’ regions that the HMM fails to capture. Surprisingly, 93% of indels are in ‘dense’ spans, yet the collective length of these spans comprises only 45% of the genome (Figure 5C(ii)). While the indel-dense regions contain nearly 50% more SNPs and repeat sequences than sparse regions, indels in dense regions outnumber those in sparse regions by more than five-fold. Based on the increased indel propensity we observed in regulatory regions (Figure 4E), we postulated that dense regions may disproportionately include regulatory regions. However, dense and sparse regions contained comparable levels of coding bases (and, by proxy, their adjacent regulatory DNA; Figure 5D). The amount of coding DNA would be a bad proxy for regulatory DNA if indel-dense spans were so short that they separated regulatory regions from their coding DNA, but we found that indel-dense spans were approximately 8.5 kb on average and often >20 kb (Additional file 1: Figure S5), thereby including many genes and their coupled regulatory elements.

Indels and their substrates (that is, repeat sequences) are enriched near replication origins. We measured indel density in 7.5 kb windows surrounding the 142 high-confidence replication origins mapped across the *C. albicans* genome [30] (Figure 5E). Indel density peaked at the native origin locations but fell nearly 10% when the origin positions were offset *in silico*. Interestingly, the density of repeat sequences also peaks near replication origins and decays to baseline levels within a few kilobases (Figure 5F). Indeed, the rate of indels per repeat sequence is approximately 20% irrespective of replication-origin proximity, suggesting that the peak in indel density is largely a result of high repeat-sequence density. However, since repeats themselves can arise from serial insertions throughout evolution, the interplay between indels and repeats at replication origins is likely complex.

### A one-parameter model predicts repeat sequence abundance from indel rate

**Figure 6:**
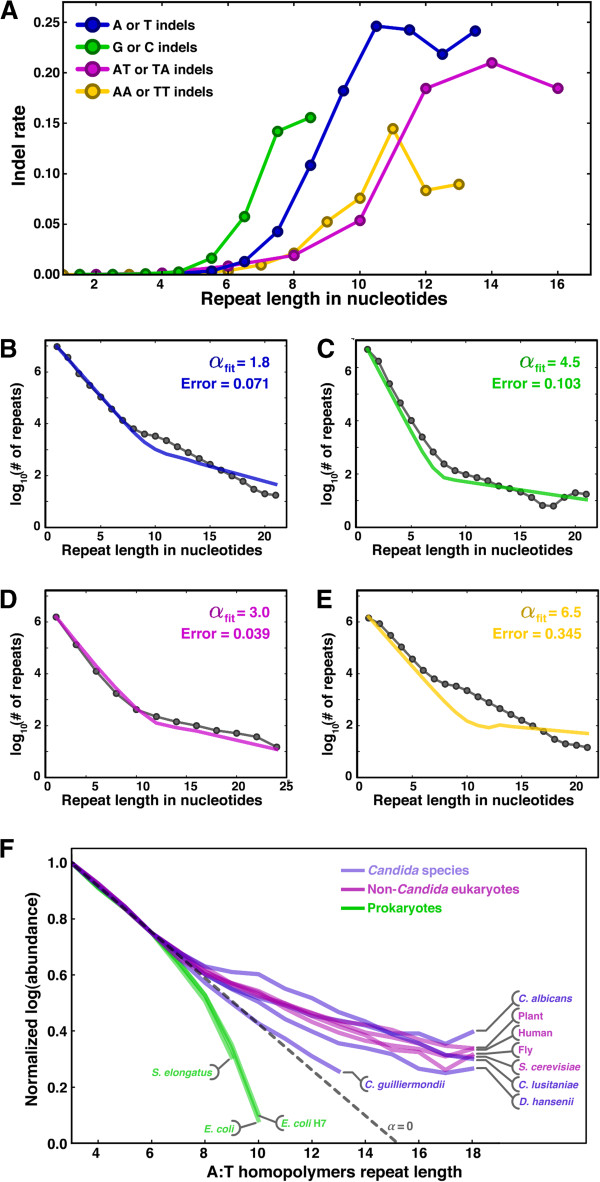
**One-parameter model reveals strong relationship between indel rate and repeat-sequence abundance. (A)** Indel rate as a function of repeat length is plotted, with coloring indicating the inserted or deleted nucleotides as shown in the legend. Repeat length is the average of the ‘reference’ and ‘indel’ read lengths; thus, for single-base indels, repeat length is ‘*x*.5’ for integer values of *x*. **(B**-**E)** Gray dotted lines show repeat-sequence abundance as a function of length for A:T homopolymers **(B**, **E)** G:C homopolymers **(C)**, and AT:TA dyad-repeats **(D)**. The colored lines show the lowest-error model fit based on the indel rates in **(A)**, with error and *α* values specified. To prevent overfitting at low repeat-length values, error is calculated as the average squared deviation in log space, not linear space. **(F)** Abundance of A:T homopolymers as a function of length in various indicated organisms. A histogram was generated for each species independently; to facilitate comparisons among species, the data were then normalized such that the abundance at length 3 is 1.0 and then scaled - to adjust for differences in genomic A:T content - such that the abundance at length 6 is 0.75. The dashed line indicates where *α* = 0.

We further explored the relationship between indels and repeat sequences by investigating the correspondence between the indel rate and the abundance of repeat sequences throughout the genome. We plotted the indel rate as a function of repeat length and noticed a dramatic increase when the repeat contains between five and seven units, reaching a maximum within three to five additional repeat units (Figure 6A). A sharp change in slope near five to seven repeat units was also evident in the log-abundance of genomic repeat sequences as a function of length (Figure 6B-D, gray traces). The fact that the plots of both the indel rate and repeat-sequence abundance have dramatic changes at similar repeat lengths suggested that a direct causative relationship exists between the two quantities. Since the traces of repeat-unit abundance appeared to be locally linear in logarithmic space, we envisioned a simple multiplicative model for repeat abundance based on the indel rate:

$$R_{n}=R_{1}\;*\prod_{i=1}^{n-1}\left[p\left(R\right)+\alpha \;*I_{R}\left(n\right)\right]$$

where *R*_*n*_ is the number of repeats of length *n* across the genome, *p(R)* is the probability of a single repeat unit (for example, the frequency of adenine nucleotides in the genome), *I*_*R*_*(n)* is the observed indel rate for repeats of length *n*, and *α* is a scalar multiplier of the indel rate. For short repeats, indels are rare (*I*_*R*_*(n)* ~ 0), so the model predicts that the number of repeats is unbiased, dominated simply by the random probability of incorporating a single repeat unit, *p(R)*. However, once the indel rate rises, it modulates the probability of adding more repeat units: for positive values of *α*, longer repeats are more favored than random, as we observed in *C. albicans*.

This one-parameter model fits well with the data when the indel rate specifies single-unit changes. For instance, the model matches well to the abundance of homopolymers consisting of As or Ts when *I*_*R*_*(n)* was the indel rate for single units (Figure 6A, B, blue trace). The same was true for homopolymers of G and C (Figure 6C), though the model is not limited to homopolymers, since it corresponds well to the abundance of dyad repeats (for example, ‘ATATATATAT’) when *I*_*R*_*(n)* was the indel rate for AT or TA dyads (purple trace, Figure 6A, D). Each fit had a different value of *α*, though all were positive and of a similar order of magnitude, ranging from 1.8 to 4.5. The assumption of single-unit changes was an important feature of the model, since there was no value of *α* such that the indel rate for two-unit changes (for example, ‘AA’ or ‘TT’; Figure 6A, yellow trace) led to a good model fit (Figure 6E). Collectively, our model is consistent with the overabundance of long repeats in the genome arising from single-unit plasticity that is ultimately biased toward insertions over deletions (that is, positive *α*). Strikingly, positive *α* values are common across a broad range of species, but only in eukaryotes, not in prokaryotes (Figure 6F; see Discussion).

## Discussion

Here we report the assembly of a completely phased diploid genome sequence for the standard *C. albicans* laboratory reference strain. We extensively revised the number of SNPs from prior assemblies [16,17], in total phasing 65,787 SNPs resulting in a nearly two-fold improvement in haplotype resolution over array-based efforts [18]. This increase in phasing resolution facilitates the detection of allele-specific phenomena by allowing comparison of allele-specific reads across multiple SNPs and subsequent pooling of the signal. Finally, we additionally identified and phased 6,103 short indels, finding that their distribution throughout the genome is significantly non-uniform.

A fully phased diploid genome for a unicellular model organism like *C. albicans* has the potential to greatly advance our ability to identify sequence determinants underlying various cellular phenomena involving nucleic acids (for example, nucleosome positioning, expression levels, secondary structure, and so on). Such determinants can be elucidated because measuring differences between thousands of allelic pairs provides a broad scope of sequence variants while simultaneously facilitating the attribution of expression differences to particular sequence features. In other words, since each allelic pair has only a few polymorphisms, phenomenological deviations between alleles can be more easily attributed to specific sequence features than is possible when comparing totally different genes. Due to the technical difficulties associated with mapping haplotypes, allele-specific measurements have predominantly relied on inbred strains or unnatural hybrid diploid organisms, where viable haploids could be sequenced prior to hybrid construction such that the respective haplotypes were known [22,31-33]. Since *C. albicans* is a natural organism that is almost exclusively diploid and frequently heterozygous, its alleles may have evolved complex and physiologically relevant interactions that would not have developed in a multi-species hybrid or highly inbred population.

We found that indels are spatially clustered throughout the genome, with nearly 93% of indels in multikilobase spans that collectively comprise only 45% of the genome. In that both indel-dense and indel-sparse regions contain a similar level of coding sequence, it seems unlikely that the disparity in indels arises from gross differences in sequence context (for example, coding sequences versus telomeres). We speculate that these spans could result from regions undergoing loss-of-heterozygosity (LOH) events, which are frequently observed in *C. albicans*; however, such events typically involve whole chromosomes (or, at the very least, large chromosomal regions), and it thus remains unclear whether there is an alternative LOH mechanism that occurs on a shorter length scale that can effectively erase indels through homozygosis.

The subtle increase in indel and repeat-sequence density that we observe near replication origins poses an interesting conundrum. In particular, it is not actually clear whether indels are favored near replication origins over an evolutionary timescale. At first, it seems that there are simply more indels near origins because there are also more repeats, arguing that indels have no higher propensity to occur near origins than elsewhere. However, since indels may be the driving force behind the creation of repeats in the first place - a possibility reinforced by our one-parameter model - indels may indeed be more likely near origins. For instance, one could imagine that a DNA polymerase prone to introducing indels is not as processive as ordinary polymerases, thus leading to an indel bias (and potentially a repeat bias) near origins. Ultimately, it is nontrivial to resolve this problem since the LOH events that occur in *C. albicans* erase indels but preserve repeats.

Due to their role in positioning nucleosomes and relevance to human disease, long repeats in eukaryotic genomes have been the topic of many experimental and quantitative modeling studies [26,29,34-36]. Here we find that a very simple model based on the indel rate for single repeat units can predict the abundance of repeats across the *C. albicans* genome. Though we cannot exclude the possibility that this correspondence is purely coincidental, it seems unlikely that one of the few mechanisms that locally alters the length of DNA (that is, indels) is not involved in the generation of DNA sequences whose length is the chief determinant of their function (that is, repeats). A causative relationship is further supported by the correlation between sharp increases in the indel rate and marked increases in repeat abundance for both homopolymers and dyad repeats.

Interestingly, repeat abundance in nearly all eukaryotes we investigated (including *C. albicans* and *Saccharomyces cerevisiae*) suggests that the net effect of indels is to lengthen repeats (that is, the indel-rate multiplier *α* is positive) (Figure 6F). The simplest explanation of this observation is that insertions occur more frequently than deletions. However, at least in *S. cerevisiae*, where the balance between insertions and deletions has been explored directly [28,29,37], deletions far outnumber insertions over laboratory timescales. It is likely that the difference in timescales explains this paradox: the observed evolutionary bias is a convolution of both mechanistic forces in the short term and selective biases in the long term; thus, while deletions may occur more frequently than insertions, selection could favor insertions, leading to their preferential fixation over time. It is also possible that the selective force stems largely from nucleosome positioning, since the prokaryotes we explored lack both nucleosomes and, critically, an overabundance of long repeats (Figure 6F). In fact, the *Escherichia coli* and *Synechococcus elongatus* genomes have far fewer long repeats than expected by chance (that is, they have negative *α* values; Figure 6F), consistent with a mechanistic bias toward deletions that is not countered by an opposing selective bias toward insertions. Further insight into the molecular determinants of the sign and magnitude of *α* may be gained by analyzing *Candida guilliermondii*, which is the only organism we found with *α* near zero. Since the *Candida* species other than *C. albicans* in Figure 6F were all sequenced by the same institute [15], we do not expect that the observation of *α* ~ 0 for *C. guilliermondii* is an artifact of the sequencing platform. The next two nearest neighbors in the *Candida* phylogeny - *Debaryomyces hansenii* and *Candida lusitaniae* - may be additionally informative, as they have progressively higher *α* values, with *α* in *D. hansenii* slightly below the range of *α* values in other eukaryotes and *α* in *C. lusitaniae* within the range.

## Conclusions

We have shown that the fully phased *C. albicans* genome reveals phenomena that are both expected, such as allele-specific expression, and unexpected, such as indel clustering. We anticipate that the higher degree of genomic resolution provided here will empower not only future researchers of this important model organism, but also those who study allele-specific traits and expression characteristics more generally.

## Methods

### Strains

In addition to the SC5314 wild-type strain, the following homozygosed strains - all from [38] and generously provided by Judith Berman - were sequenced: AF9318-1 (1AA), AF3990 (2AA), RBY_10-10 (3AA), RBY_E-6 (4AA), AF4448_SC5314_MTLa (5AA), RBY10-12 (6AA), RBY_9-3sm (7AA), YJB10699 (RAA), and YJB10698 (RBB). Sequencing and SNP detection on the ostensibly ‘RBB’ strain revealed that it was not, in fact, homozygous for the B homolog of chromosome R (Figure 2A, B); nonetheless, the extra sequencing information from this strain enhanced polymorphism identification.

### Library preparation and sequencing

Genomic DNA was prepared from saturated overnight cultures from single colonies grown in YPD. Cell pellets were ruptured by vortexing with glass beads; DNA was extracted using phenol/chloroform/isoamylalcohol (25:24:1) and then precipitated in isopropanol and sodium acetate. After ethanol washing, DNA was resuspended in TE + RNase. Library construction methods were previously described [39]. Libraries were sequenced with a 36 nucleotide paired-end kit on an Illumina Genome Analyzer IIx.

### Read alignment

For SNP identification, reads were mapped to the Assembly 21 reference genome using Bowtie (v1.0) [19], allowing up to three mismatches. Reads that failed to align using Bowtie were subsequently reprocessed using custom scripts written in PHP. Specifically, a localized best-match alignment was performed on unaligned reads with a paired-end read that aligned correctly (Additional file 1: Figure S2A). The localized alignment on the unaligned read was performed at all offsets within a window 200 to 800 bases away from the correctly aligned position of the paired end. If the unaligned read matched at least 30 bases (that is, no more than 6 mismatches) at a single offset and did not match more than 18 bases at any other offset in the window, then the read was designated as having aligned at the given offset. For indel identification, the reads that failed to align using both Bowtie and the window strategy described above were remapped using BWA (v0.5.9-r16) [40]. Raw sequencing data are available from the NIH SRA (BioProject SRP022363).

### Allele-specific expression analysis

Reads from [21] - accession number SRA020929, runs SRR060102, SRR060124, SRR060125, SRR060126, SRR060099, SRR060100, SRR060101, SRR064145, and SRR064146 - were mapped to the phased genome assembly (see Additional files 3 and 4 for FASTA files) using Bowtie and allowing three mismatches. Custom software written in C parsed the alignment file, finding reads that contained SNPs and designating them based on whether they mapped to the A or B allele. Reads that mapped within 100 nucleotides upstream of a gene’s start codon and 100 nucleotides downstream of its stop codon were attributed to that gene (see Additional file 2 for allele-specific expression counts for all alleles). Candidates for NMD analysis were selected based on the following criteria: (1) to avoid potential gene-start annotation errors at the 5′ end, the length of the shorter allele must be at least 20% of the longer allele; (2) to ensure that the downstream sequence elements that help to elicit NMD [41] are present, the length of the shorter allele must be less than 80% of the length of the longer allele; (3) to ensure that both alleles are expressed, each must have at least five allele-specific reads in the RNA-seq dataset; (4) to exclude dubious ORFs from assembly 21, the reading frame must start with ATG or a near-cognate start codon (for example, AGG, ACG, and so on).

### Hidden Markov model

The two-state HMM (Figure 5) was fit using an implementation of the Viterbi algorithm [42] in Python.

## Abbreviations

HMM: Hidden Markov model; indel: Insertion/deletion; LOH: Loss-of-heterozygosity; NMD: Nonsense-mediated decay; ORF: Open reading frame; PTC: Premature termination codon; SNP: Single-nucleotide polymorphism.

## Competing interests

The authors declare that they have no competing interests.

## Authors’ contributions

DM, JSW, and GS designed the study. DM prepared samples, and KS finished library preparation and performed DNA sequencing. DM performed analysis of the data and drafted the manuscript. JSW and GS offered insights into the analysis and edited the manuscript. All authors read and approved the final manuscript.

## Supplementary Material

Additional file 1 Figures S1 to S5, Table S1. Click here for file

Additional file 2 **Table listing characteristics of *C. albicans* ORFs, including coordinates in phased assembly, allele-specific expression levels, number of SNPs and indels in coding region, and number of indels in promoter.** Click here for file

Additional file 3 Phased FASTA file (SNPs only). Click here for file

Additional file 4 Phased FASTA file (SNPs plus indels). Click here for file

## References

[B1] Browning SR Browning BL Haplotype phasing: existing methods and new developments Nat Rev Genet 2011 14 703 714 21921926 10.1038/nrg3054 PMC3217888

[B2] Lin S Chakravarti A Cutler DJ Haplotype and missing data inference in nuclear families Genome Res 2004 14 1624 1632 10.1101/gr.2204604 15256514 PMC509272

[B3] Li X Li J Haplotype reconstruction in large pedigrees with untyped individuals through IBD inference J Comput Biol 2011 14 1411 1421 10.1089/cmb.2011.0167 21923410 PMC3216110

[B4] Ma L Xiao Y Huang H Wang Q Rao W Feng Y Zhang K Song Q Direct determination of molecular haplotypes by chromosome microdissection Nat Methods 2010 14 299 301 10.1038/nmeth.1443 20305652 PMC2871314

[B5] Fan HC Wang J Potanina A Quake SR Whole-genome molecular haplotyping of single cells Nat Biotechnol 2011 14 51 57 10.1038/nbt.1739 21170043 PMC4098715

[B6] Peters BA Kermani BG Sparks AB Alferov O Hong P Alexeev A Jiang Y Dahl F Tang YT Haas J Robasky K Zaranek AW Lee J-H Ball MP Peterson JE Perazich H Yeung G Liu J Chen L Kennemer MI Pothuraju K Konvicka K Tsoupko-Sitnikov M Pant KP Ebert JC Nilsen GB Baccash J Halpern AL Church GM Drmanac R Accurate whole-genome sequencing and haplotyping from 10 to 20 human cells Nature 2012 14 190 195 10.1038/nature11236 22785314 PMC3397394

[B7] Kaper F Swamy S Klotzle B Munchel S Cottrell J Bibikova M Chuang H-Y Kruglyak S Ronaghi M Eberle MA Fan J-B Whole-genome haplotyping by dilution, amplification, and sequencing Proc Natl Acad Sci U S A 2013 14 5552 5557 10.1073/pnas.1218696110 23509297 PMC3619281

[B8] Kitzman JO Mackenzie AP Adey A Hiatt JB Patwardhan RP Sudmant PH Ng SB Alkan C Qiu R Eichler EE Shendure J Haplotype-resolved genome sequencing of a Gujarati Indian individual Nat Biotechnol 2011 14 59 63 10.1038/nbt.1740 21170042 PMC3116788

[B9] Suk E-K McEwen GK Duitama J Nowick K Schulz S Palczewski S Schreiber S Holloway DT McLaughlin S Peckham H Lee C Huebsch T Hoehe MR A comprehensively molecular haplotype-resolved genome of a European individual Genome Res 2011 14 1672 1685 10.1101/gr.125047.111 21813624 PMC3202284

[B10] Voskoboynik A Neff NF Sahoo D Newman AM Pushkarev D Koh W Passarelli B Fan HC Mantalas GL Palmeri KJ Ishizuka KJ Gissi C Griggio F Ben-Shlomo R Corey DM Penland L White RA Weissman IL Quake SR The genome sequence of the colonial chordate, Botryllus schlosseri Elife 2013 14 e00569 23840927 10.7554/eLife.00569 PMC3699833

[B11] Bennett RJ Johnson AD Mating in Candida albicans and the search for a sexual cycle Annu Rev Microbiol 2005 14 233 255 10.1146/annurev.micro.59.030804.121310 15910278

[B12] Bennett RJ Johnson AD Completion of a parasexual cycle in Candida albicans by induced chromosome loss in tetraploid strains EMBO J 2003 14 2505 2515 10.1093/emboj/cdg235 12743044 PMC155993

[B13] Forche A Alby K Schaefer D Johnson AD Berman J Bennett RJ The parasexual cycle in Candida albicans provides an alternative pathway to meiosis for the formation of recombinant strains PLoS Biol 2008 14 e110 10.1371/journal.pbio.0060110 18462019 PMC2365976

[B14] Hickman MA Zeng G Forche A Hirakawa MP Abbey D Harrison BD Wang Y-M Su C-H Bennett RJ Wang Y Berman J The “obligate diploid” Candida albicans forms mating-competent haploids Nature 2013 14 55 59 10.1038/nature11865 23364695 PMC3583542

[B15] Butler G Rasmussen MD Lin MF Santos MAS Sakthikumar S Munro CA Rheinbay E Grabherr M Forche A Reedy JL Agrafioti I Arnaud MB Bates S Brown AJP Brunke S Costanzo MC Fitzpatrick DA de Groot PWJ Harris D Hoyer LL Hube B Klis FM Kodira C Lennard N Logue ME Martin R Neiman AM Nikolaou E Quail MA Quinn J Evolution of pathogenicity and sexual reproduction in eight Candida genomes Nature 2009 14 657 662 10.1038/nature08064 19465905 PMC2834264

[B16] Jones T Federspiel NA Chibana H Dungan J Kalman S Magee BB Newport G Thorstenson YR Agabian N Magee PT Davis RW Scherer S The diploid genome sequence of Candida albicans Proc Natl Acad Sci U S A 2004 14 7329 7334 10.1073/pnas.0401648101 15123810 PMC409918

[B17] van het Hoog M Rast TJ Martchenko M Grindle S Dignard D Hogues H Cuomo C Berriman M Scherer S Magee BB Whiteway M Chibana H Nantel A Magee PT Assembly of the Candida albicans genome into sixteen supercontigs aligned on the eight chromosomes Genome Biol 2007 14 R52 10.1186/gb-2007-8-4-r52 17419877 PMC1896002

[B18] Abbey D Hickman M Gresham D Berman J High-Resolution SNP/CGH microarrays reveal the accumulation of loss of heterozygosity in commonly used Candida albicans strains G3 (Bethesda) 2011 14 523 530 2011 22384363 10.1534/g3.111.000885 PMC3276171

[B19] Langmead B Trapnell C Pop M Salzberg SL Ultrafast and memory-efficient alignment of short DNA sequences to the human genome Genome Biol 2009 14 R25 10.1186/gb-2009-10-3-r25 19261174 PMC2690996

[B20] Hoyer LL The ALS gene family of Candida albicans Trends Microbiol 2001 14 176 180 10.1016/S0966-842X(01)01984-9 11286882

[B21] Bruno VM Wang Z Marjani SL Euskirchen GM Martin J Sherlock G Snyder M Comprehensive annotation of the transcriptome of the human fungal pathogen Candida albicans using RNA-seq Genome Res 2010 14 1451 1458 10.1101/gr.109553.110 20810668 PMC2945194

[B22] Gregg C Zhang J Weissbourd B Luo S Schroth GP Haig D Dulac C High-resolution analysis of parent-of-origin allelic expression in the mouse brain Science 2010 14 643 648 10.1126/science.1190830 20616232 PMC3005244

[B23] DeVeale B van der Kooy D Babak T Critical evaluation of imprinted gene expression by RNA-Seq: a new perspective PLoS Genet 2012 14 e1002600 10.1371/journal.pgen.1002600 22479196 PMC3315459

[B24] Kelsey G Bartolomei MS Imprinted genes … and the number is? PLoS Genet 2012 14 e1002601 10.1371/journal.pgen.1002601 22479197 PMC3315456

[B25] Chang Y-F Imam JS Wilkinson MF The nonsense-mediated decay RNA surveillance pathway Annu Rev Biochem 2007 14 51 74 10.1146/annurev.biochem.76.050106.093909 17352659

[B26] Segal E Widom J Poly(dA:dT) tracts: major determinants of nucleosome organization Curr Opin Struct Biol 2009 14 65 71 10.1016/j.sbi.2009.01.004 19208466 PMC2673466

[B27] Raveh-Sadka T Levo M Shabi U Shany B Keren L Lotan-Pompan M Zeevi D Sharon E Weinberger A Segal E Manipulating nucleosome disfavoring sequences allows fine-tune regulation of gene expression in yeast Nat Genet 2012 14 743 750 10.1038/ng.2305 22634752

[B28] Zanders S Ma X Roychoudhury A Hernandez RD Demogines A Barker B Gu Z Bustamante CD Alani E Detection of heterozygous mutations in the genome of mismatch repair defective diploid yeast using a Bayesian approach Genetics 2010 14 493 503 10.1534/genetics.110.120105 20660644 PMC2954485

[B29] Gragg H Harfe BD Jinks-Robertson S Base composition of mononucleotide runs affects DNA polymerase slippage and removal of frameshift intermediates by mismatch repair in Saccharomyces cerevisiae Mol Cell Biol 2002 14 8756 8762 10.1128/MCB.22.24.8756-8762.2002 12446792 PMC139878

[B30] Koren A Tsai H-J Tirosh I Burrack LS Barkai N Berman J Epigenetically-inherited centromere and neocentromere DNA replicates earliest in S-phase PLoS Genet 2010 14 e1001068 10.1371/journal.pgen.1001068 20808889 PMC2924309

[B31] Tirosh I Reikhav S Levy AA Barkai N A yeast hybrid provides insight into the evolution of gene expression regulation Science 2009 14 659 662 10.1126/science.1169766 19407207

[B32] Khan Z Bloom JS Amini S Singh M Perlman DH Caudy AA Kruglyak L Quantitative measurement of allele-specific protein expression in a diploid yeast hybrid by LC-MS Mol Syst Biol 2012 14 602 22893000 10.1038/msb.2012.34 PMC3435501

[B33] Zhang X Borevitz JO Global analysis of allele-specific expression in Arabidopsis thaliana Genetics 2009 14 943 954 10.1534/genetics.109.103499 19474198 PMC2728882

[B34] Tran HT Keen JD Kricker M Resnick MA Gordenin DA Hypermutability of homonucleotide runs in mismatch repair and DNA polymerase proofreading yeast mutants Mol Cell Biol 1997 14 2859 2865 9111358 10.1128/mcb.17.5.2859 PMC232138

[B35] Kelkar YD Strubczewski N Hile SE Chiaromonte F Eckert KA Makova KD What is a microsatellite: a computational and experimental definition based upon repeat mutational behavior at A/T and GT/AC repeats Genome Biol Evol 2010 14 620 635 10.1093/gbe/evq046 20668018 PMC2940325

[B36] Kelkar YD Tyekucheva S Chiaromonte F Makova KD The genome-wide determinants of human and chimpanzee microsatellite evolution Genome Res 2008 14 30 38 18032720 10.1101/gr.7113408 PMC2134767

[B37] Lang GI Murray AW Estimating the per-base-pair mutation rate in the yeast Saccharomyces cerevisiae Genetics 2008 14 67 82 10.1534/genetics.107.071506 18202359 PMC2206112

[B38] Legrand M Forche A Selmecki A Chan C Kirkpatrick DT Berman J Haplotype mapping of a diploid non-meiotic organism using existing and induced aneuploidies PLoS Genet 2008 14 e1 10.1371/journal.pgen.0040001 18179283 PMC2174976

[B39] Schwartz K Wenger JW Dunn B Sherlock G APJ1 and GRE3 homologs work in concert to allow growth in xylose in a natural Saccharomyces sensu stricto hybrid yeast Genetics 2012 14 621 632 10.1534/genetics.112.140053 22426884 PMC3374322

[B40] Li H Durbin R Fast and accurate short read alignment with Burrows-Wheeler transform Bioinformatics 2009 14 1754 1760 10.1093/bioinformatics/btp324 19451168 PMC2705234

[B41] Peltz SW Brown AH Jacobson A mRNA destabilization triggered by premature translational termination depends on at least three cis-acting sequence elements and one trans-acting factor Genes Dev 1993 14 1737 1754 10.1101/gad.7.9.1737 8370523

[B42] Viterbi A Error bounds for convolutional codes and an asymptotically optimum decoding algorithm Information Theory 1967 14 260 269

