# Supplementary material for: Assembly of a phased diploid Candida albicans genome facilitates allele-specific measurements and provides a simple model for repeat and indel structure
Source: Genome Biol. 2013 Sep 11;14(9):R97. doi: 10.1186/gb-2013-14-9-r97 (PMC4054093; doi:10.1186/gb-2013-14-9-r97)
Supplement: Additional file 1 — Figures S1 to S5, Table S1. [file gb-2013-14-9-r97-S1.pdf]

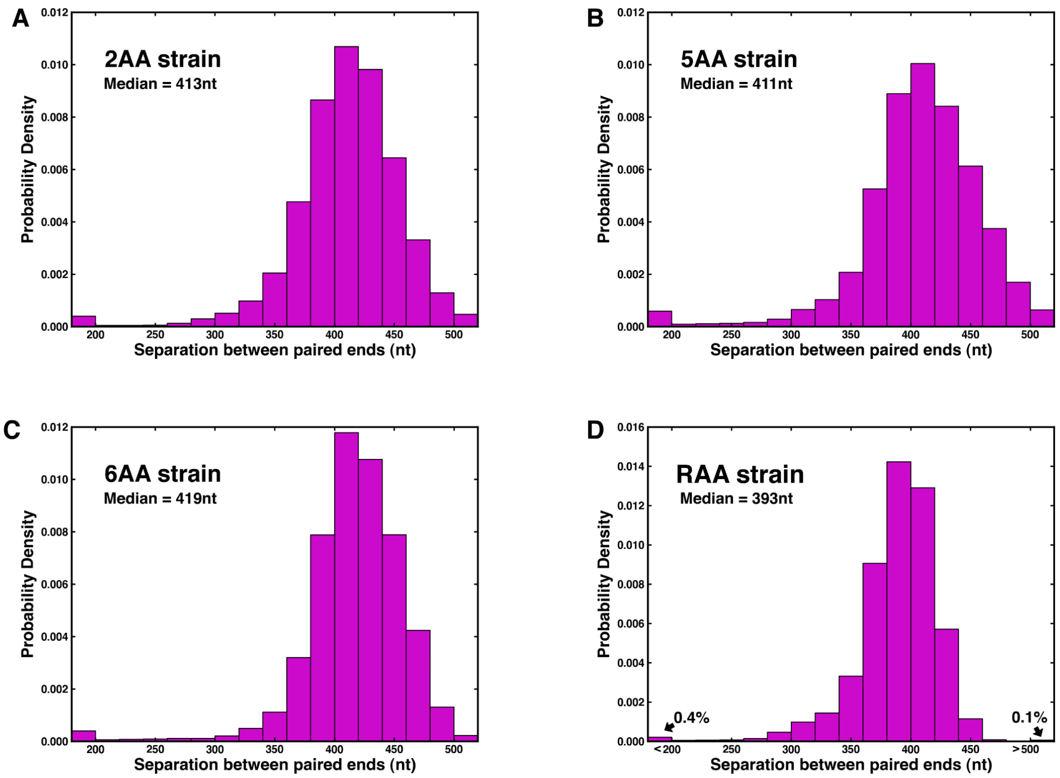

Figure S1—Paired-end fragment size tended to be between 300 and 500 nucleotides.

For four representative strains as indicated **(A-D)**, the distance between the alignment positions for each end of a paired end read is compiled into a histogram.

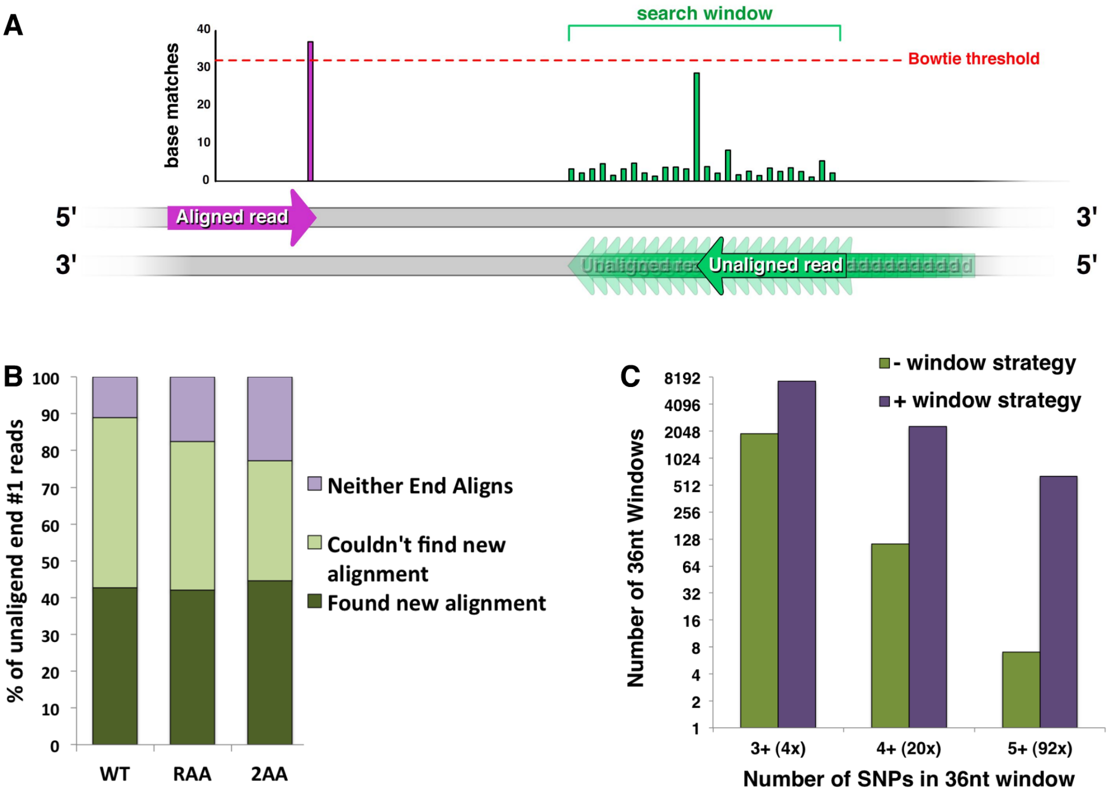

Figure S2—Originally unaligned reads were mapped to SNP-dense regions using information from an aligned paired-end read.
(A) When a read failed to align using Bowtie due to too many mismatches (shown in green), we counted the number of matches within a search window, which was 200 to 800 nucleotides away from the aligned paired-end (shown in purple). As schematized, there was frequently a single position with far more matches than background, and we assigned the position of the read to this position, as specified in Methods. **(B)** For three representative strains, approximately 80% of unaligned reads had a paired-end that aligned to the genome (non-purple portions). Of these, nearly 50% (dark green) could be aligned to the genome using the window strategy depicted in **(A)**. **(C)** Using the window strategy in **(A)** significantly increased the number of SNP-dense regions identified (compare purple and green bars). For example, more than 500 non-overlapping 36nt-windows contain 5+ SNPs when using the window strategy, a 92-fold increase over the few such windows identified without the window strategy.

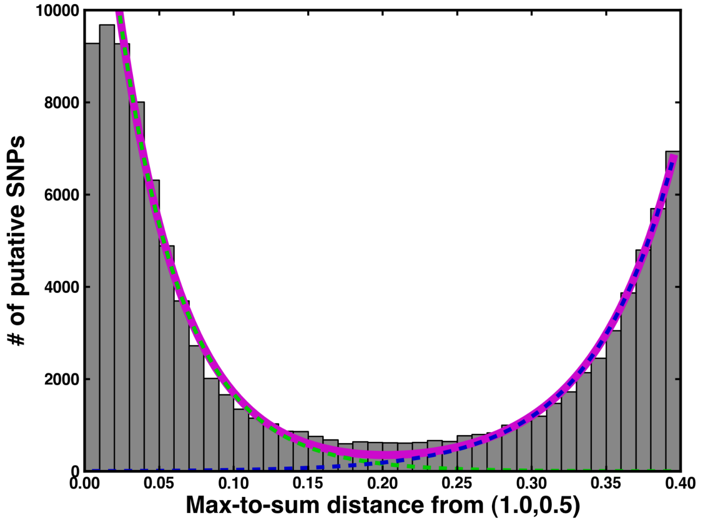

Figure S3—A cutoff for SNP identification was selected based on the marginal error rate.
For all points in Figure 2C, the Euclidean distance from (1.0,0.5) was calculated and compiled into a histogram (the majority of non-SNP points are excluded since the x-axis was truncated at a distance of 0.4, and most non-SNPs have distances >0.4). The resulting histogram was very well approximated as the sum of two exponential distributions, one in green indicating true SNPs, and the other in blue representing false SNPs (the sum is in pink). The distance cutoff of 0.195 was selected since that is the distance at which the probabilities of true versus false SNPs are equal.

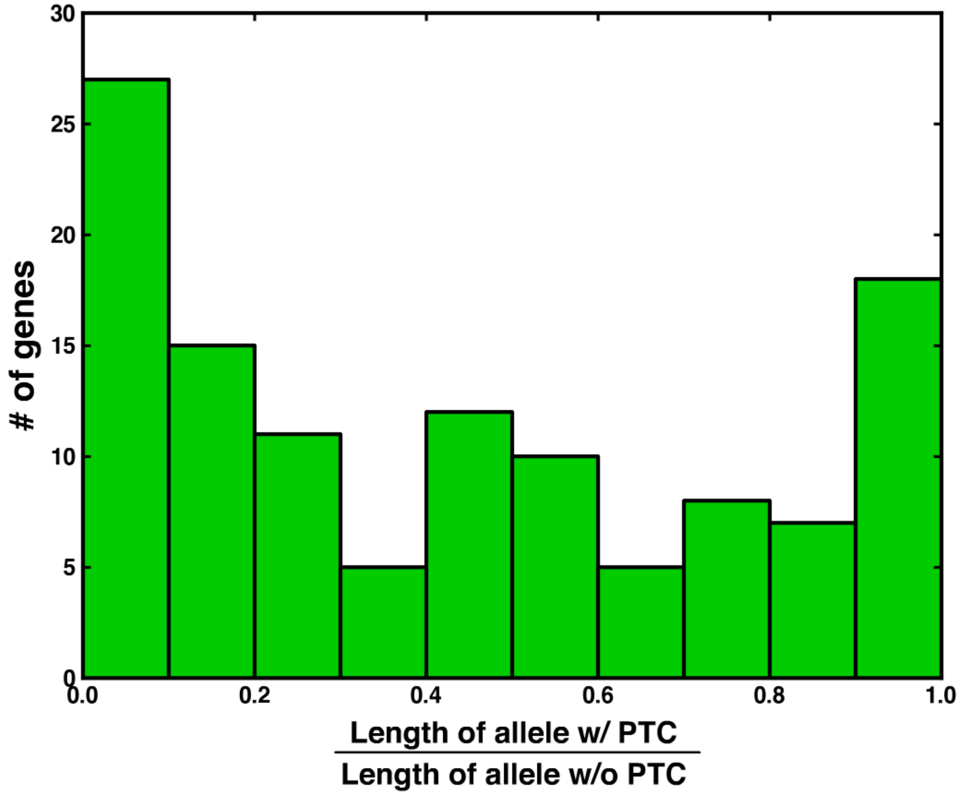

Figure S4—PTCs are enriched in at the 5' and 3' ends of genes.
The lengths of coding regions for alleles bearing PTCs were divided by the respective lengths of the counterpart alleles lacking PTCs, and these ratios were compiled into a histogram.

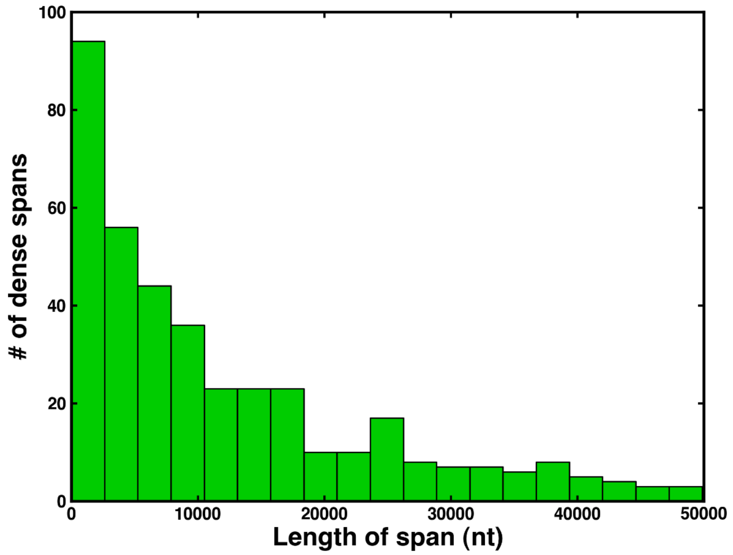

Figure S5—Indel-dense regions frequently span tens of kilobases.
The length of all “dense” regions, as depicted in Figure 5C, is compiled into a histogram.

GFF Index	Gene_Name	Longer Allele	Highly expressed allele	A_Counts	B_Counts	Allelic_Bias_%
1163	orf19.4482	***A***	***A***	22	6	366.7
555	orf19.1774	A	B	34	119	350.0
1556	orf19.4736	***A***	***A***	125	37	337.8
746	orf19.1078	***B***	***B***	154	358	232.5
845	orf19.1557	***B***	***B***	44	81	184.1
723	orf19.6025	***B***	***B***	22	37	168.2
2134	orf19.2995	B	A	89	53	167.9
612	orf19.2204.2	***A***	***A***	26	16	162.5
2818	orf19.1356	***B***	***B***	25	40	160.0
4098	orf19.4767	***B***	***B***	36	56	155.6
5172	orf19.4511	***B***	***B***	27	41	151.9
665	orf19.3686	B	A	237	163	145.4
2986	orf19.2227	***A***	***A***	55	42	131.0
4262	orf19.1508	***B***	***B***	9	11	122.2
730	orf19.35.1	***B***	***B***	408	495	121.3
4078	orf19.4981	A	B	50	60	120.0
3636	orf19.5576	A	B	170	199	117.1
4022	orf19.1365	A	A	11	10	110.0
2118	orf19.4702	A	A	69	63	109.5
1174	orf19.3784	A	A	12	11	109.1
2531	orf19.4321	A	A	14	13	107.7
837	orf19.5079	A	B	445	447	100.4

Table S1—Alleles with PTCs have lower levels than their counterpart alleles. The genes included in NMD analysis (see Methods) are ranked according to their allelic bias. Light-gray shading indicates genes with less than 20% allelic bias, and red letters indicate genes where the differences between allele length and expression are consistent with NMD.
